# A Case of Left Ventricular Pseudoaneurysm as a Complication of Late-Presenting ST-Segment Elevation Myocardial Infarction

**DOI:** 10.7759/cureus.60026

**Published:** 2024-05-10

**Authors:** Andrew Engel-Rodriguez, Jose Escabi-Mendoza, Victor H Molina-Lopez, Natalie Engel-Rodriguez, Marilee Tiru-Vega

**Affiliations:** 1 Internal Medicine, VA (Veterans Affairs) Caribbean Healthcare Systems, San Juan, PRI; 2 Cardiovascular Disease, VA (Veterans Affairs) Caribbean Healthcare Systems, San Juan, PRI; 3 Cardiology, VA (Veterans Affairs) Caribbean Healthcare Systems, San Juan, PRI; 4 Internal Medicine, San Juan Bautista School of Medicine, Caguas, PRI

**Keywords:** radiographic sign, echocardiogram, cardiac imaging, st-elevation myocardial infarction (stemi), hiv, intra cardiac thrombus, ventricular tachycardia (vt) storm, cardiac pseudoaneurysm

## Abstract

This case report delineates the clinical trajectory and management strategies of a 59-year-old Hispanic male diagnosed with a left ventricular pseudoaneurysm (LVPA) following a delayed presentation of ST-segment elevation myocardial infarction (STEMI), for which reperfusion treatment was not administered. Initially, an echocardiogram demonstrated an extensive anterolateral myocardial infarction, severe left ventricular systolic dysfunction, and an early-stage left ventricular apical aneurysm with thrombus, leading to the initiation of warfarin. Metabolic myocardial perfusion imaging via positron emission tomography indicated a substantial myocardial scar without viability, guiding the decision against revascularization. Post discharge, the patient, equipped with a wearable cardioverter defibrillator for sudden cardiac death prevention, experienced symptomatic ventricular tachycardia, which was resolved with defibrillator shocks. Subsequent imaging revealed an acute LVPA adjacent to the existing left ventricular aneurysm. Given the high surgical risk, conservative management was elected, resulting in thrombosis and closure of the pseudoaneurysm after two weeks. The patient eventually transitioned to home hospice, surviving an additional five months. This report underscores the complexities and therapeutic dilemmas in managing post-MI LVPA patients who are ineligible for surgical intervention.

## Introduction

Mechanical complications following an acute myocardial infarction (AMI) represent some of the most severe clinical challenges in cardiology, encompassing left ventricular (LV) free wall rupture (FWR), LV pseudoaneurysm (LVPA), ventricular septal rupture, and papillary muscle rupture. These complications are notably more prevalent following ST-segment elevation myocardial infarction (STEMI) [1-3]. The advent of early percutaneous coronary intervention (PCI) as the standard of care for acute occlusive myocardial reperfusion has significantly reduced the incidence of these mechanical complications. Historically, such complications occurred in 6% of cases between 1977 and 1982 before widespread reperfusion therapy. This rate declined to a mere 0.27% from 2003 to 2015, paralleling the establishment of primary PCI and dedicated STEMI care systems, although the associated in-hospital mortality remains high at 42% [4]. Despite this progress, delays in the recognition and treatment of AMI persist, particularly evident in the aftermath of the coronavirus disease 2019 (COVID-19) pandemic, where fears of hospital-acquired infections have deterred timely patient presentations, maintaining a residual risk of mechanical complications [5].

LVPA is an uncommon form of cardiac rupture characterized by an incomplete or contained rupture, typically sealed by scar tissue or an adherent pericardium, without the presence of endocardium or myocardium typical of a true LV aneurysm [1]. The rupture is precipitated by the loss of myocardial integrity following a transmural infarction and exacerbated by increased LV pressure and wall stress [1]. Unlike FWR, which often results in cardiac tamponade and early mortality within the first days post AMI [1-3], the presentation of LVPA can be delayed, thereby altering its clinical management and prognosis. Early, aggressive surgical or percutaneous interventions remain the preferred treatment approach, reflecting the strategy for managing FWR.

This report focuses on the clinical presentations, diagnostic strategies, and management approaches for LVPA post AMI. While LVPA is rare in the context of STEMI managed with timely reperfusion, it remains a critical consideration for patients presenting late or without reperfusion therapy. Despite its infrequency, the potential for LVPA underscores the need for heightened vigilance among healthcare providers, especially when faced with patients exhibiting nonspecific cardiopulmonary symptoms and characteristic imaging findings post AMI.

## Case presentation

A 59-year-old Hispanic male, with a past medical history of HIV, cryptococcal meningitis treated with a ventricular-peritoneal shunt, fluconazole prophylaxis, hypertension, hypothyroidism, and chronic kidney disease stage IIIa, presented to the emergency department (ED) with retrosternal chest pain. The pain, characterized as burning and pressure-like in quality and rated 5/10 in intensity (numerical rating pain scale), radiated to the neck and left arm and was associated with shortness of breath and diaphoresis that had initially appeared three to four days before his presentation. The patient had initially disregarded the chest pain but sought medical attention due to progressive worsening of shortness of breath and fatigue. Home medications were amlodipine 2.5 mg daily, aspirin 81 mg daily, darunavir ethanolate 600 mg twice a day, emtricitabine 200 mg every other day, fenofibrate 143 mg daily, levothyroxine 0.075 mg daily, metoprolol tartrate 12.5 mg twice a day, fluconazole 200 mg daily, raltegravir 400 mg twice a day, ritonavir 100 mg twice a day, and stavudine 40 mg daily. He was compliant with home medications. He was 68 inches tall and weighed 137 pounds.

**Figure 1:**
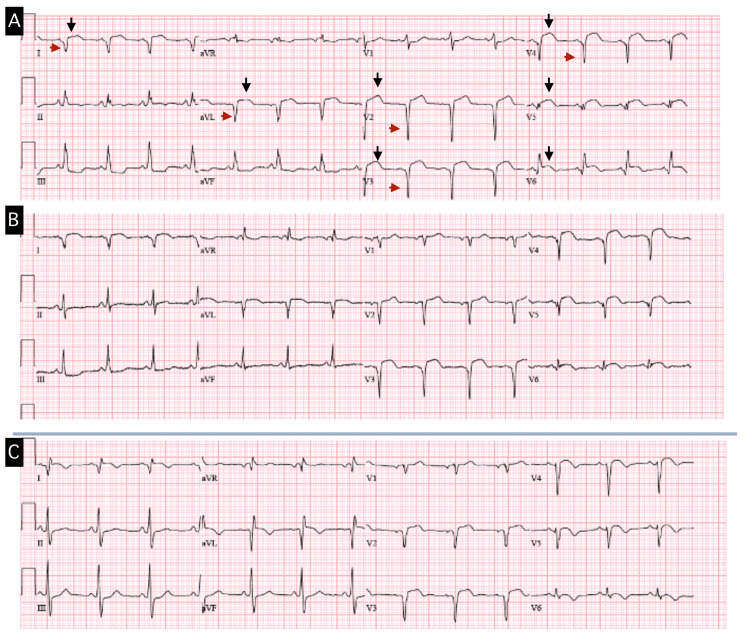
Admission electrocardiogram A. First admission ECG: Anterolateral ST-segment elevations in leads V2-V6, I, and aVL (black arrows), in the presence of pronounced QS-waves without residual R-wave in leads V2-V4, 1, and aVL (red arrows), suggestive of a late presenting transmural myocardial infarction scar. B. First admission follow-up ECG: Anterolateral ST-segment elevations and QS waves at the same leads. No significant changes. C. Second admission ECG (with pseudoaneurysm): Persistent anterolateral ST-segment elevations and QS waves at the same leads. No significant changes.

Upon ED evaluation, the patient was hemodynamically stable and afebrile. Physical examination found the patient speaking in complete sentences, with no jugular venous distension, no hepatojugular reflux, symmetric chest expansion, and no peripheral edema. Lung auscultation revealed bibasilar inspiratory rales. Cardiac examination showed a regular rate and rhythm, normal heart sounds, and no audible murmurs. His presenting electrocardiogram showed anterolateral ST-segment elevations in leads V2-V6, I, and aVL (ranging from 2-3mm), along with deep QS waves in the same leads (except V5-V6 that have R-wave present), indicative of a late-presenting anterolateral STEMI (Figure 1A). Cardiac troponins were elevated with a decreasing trend of 1.8, 1.76, and 1.68 ng/ml at 0/4/8 hours, respectively, against a normal CK-MB value of 4.5 ng/ml.

Cardiology consultation included a limited bedside two-dimensional transthoracic echocardiogram (TTE), which revealed severe anterolateral mid-apical wall akinesia to dyskinesia with an estimated LV ejection fraction (LVEF) of 25%. Given no active chest pain and the ECG and troponin findings, he was diagnosed with subacute late-presenting transmural anterolateral STEMI (>48 hours). No emergent catheterization for primary PCI was indicated, as the preferred reperfusion strategy for patients presenting with less than 12 hours of STEMI evolution was not applicable.

He was admitted to the coronary intensive care unit for close monitoring of potential post-myocardial infarction complications and received goal-directed medical therapy for acute coronary syndrome and heart failure with reduced ejection fraction (HFrEF). He was provided full heparin anticoagulation as a bridge to warfarin due to the presence of an echogenic mobile structure less than 1 cm in size in the apical region, suggestive of thrombus formation, and adjustments to his beta-blocker therapy for paroxysmal atrial fibrillation rate control. A viability study with positron emission tomography and computed tomography (PET/CT) assessed the extent of myocardial scar tissue, confirming a lack of viable myocardium and guiding further management towards conservative strategies. Prior to discharge, his medical therapy was optimized, including dual antiplatelet therapy, high-intensity statins, beta-blocker, sacubitril/valsartan, sodium-glucose cotransporter-2 (SGLT2) inhibitors, and loop diuretics, complemented by a wearable cardioverter-defibrillator (WCD) for primary prevention of sudden cardiac death.

**Figure 2:**
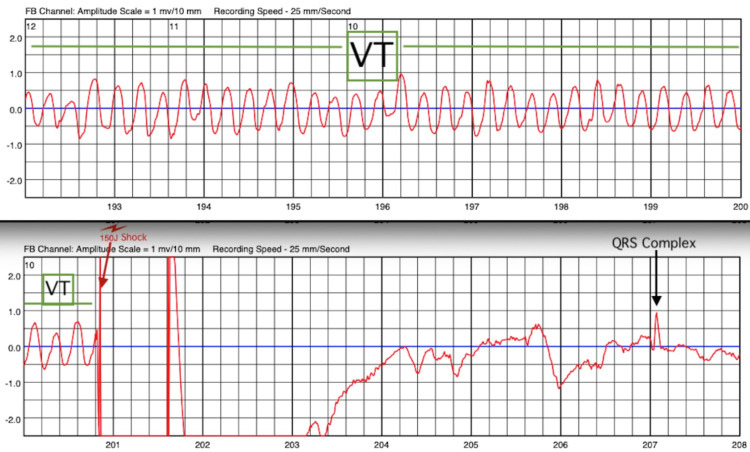
Device interrogation The wearable cardioverter defibrillator report showed episodes of sustained ventricular tachycardia (VT) (green line), which was appropriately shocked by 150 joules (red arrow), successfully aborting sudden cardiac death. QRS complexes (black arrow) returned following device discharge.

**Figure 3:**
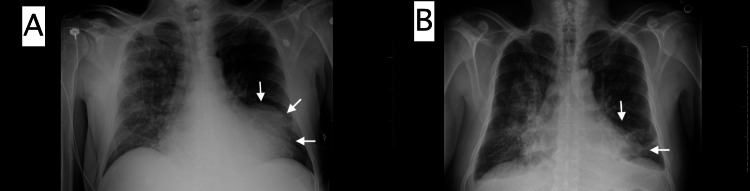
Chest radiographs A: Chest radiograph with new left ventricle border configuration (white arrow). B: Previous chest radiograph with original left ventricle border configuration (white arrow).

**Figure 4:**
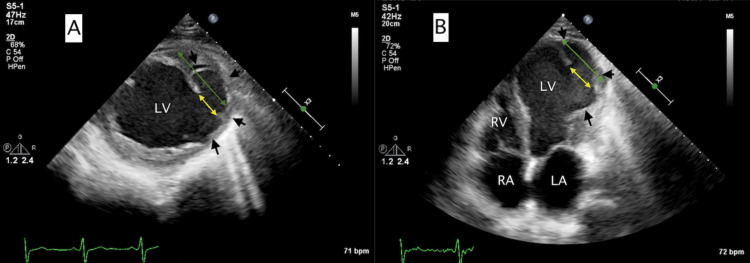
Transthoracic echocardiogram with pseudoaneurysm Echocardiogram unveiled a severely reduced ejection fraction of less than 15% and a left ventricular pseudoaneurysm upon the mid-anterolateral wall with a narrow neck of a max diameter of 2 cm (yellow arrows), corresponding to the borders of the ruptured wall, compared to a wider aneurysmal sac size of approximately 6-7 cm (green arrows). A: Two-dimensional transthoracic echocardiogram left ventricular short axis view with left ventricular pseudoaneurysm (black arrows). B: Two-dimensional transthoracic echocardiogram four-chamber view with left ventricular pseudoaneurysm (black arrows) LV: left ventricle, LA: left atrium, RV: right ventricle, RA: right atrium

Three days post discharge, the patient experienced multiple shocks from his WCD due to ventricular tachycardia (VT) triggered by chest discomfort and dizziness. Device interrogation confirmed three episodes of VT (Figure 2). Following this, he was readmitted for heart failure exacerbation and arrhythmia management, including noninvasive positive pressure ventilation and diuretic treatment. His chest X-ray on readmission revealed a new left ventricle border bulging configuration compared to the chest X-ray obtained two weeks prior (Figure 3), suggesting a possible LVA versus an LVPA. A follow-up echocardiogram confirmed the presence of an LVPA with a narrow orifice diameter of 2 cm and sac size of approximately 6-7 cm (Figure 4), with evidence of low-velocity blood flow and thrombus within the sac (Video 1). 

**Video 1:** Left ventricular pseudoaneurysm viewed with transthoracic echocardiography from a parasternal short-axis view Transthoracic echocardiography from a parasternal short axis view showing a dilated left ventricle (LV) with a severe systolic dysfunction. It also illustrates the neck of the LV pseudoaneurysm, corresponding to the borders of the ruptured anterolateral wall and leading to a contained extra-cardiac pseudoaneurysm (PA) within the pericardium. The video also shows spontaneous echo contrast noted both in the LV chamber and the PA, most significantly in the latter, with flow communicating through the neck of the PA.

The clinical course was complicated by ongoing VT. This arrhythmia necessitated intensive rhythm management strategies, including the use of electric defibrillation and pharmacologic therapy with intravenous amiodarone followed by lidocaine, which was later transitioned to oral mexiletine. The holistic management approach included optimizing heart failure medications. His medications were atorvastatin 40 mg daily, metoprolol succinate 37.5 mg daily, lisinopril 2.5 mg daily, empagliflozin 12.5 mg daily, amiodarone 200 mg daily, digoxin 0.125 mg daily, mexiletine 150 mg twice a day, furosemide 40 mg daily, and isosorbide mononitrate 30 mg daily. Warfarin was discontinued due to concerns about the progression of LVPA. Vital signs on this regimen include a heart rate of 58-71 bpm and blood pressure of 93/62-131/70 mmHg.

**Figure 5:**
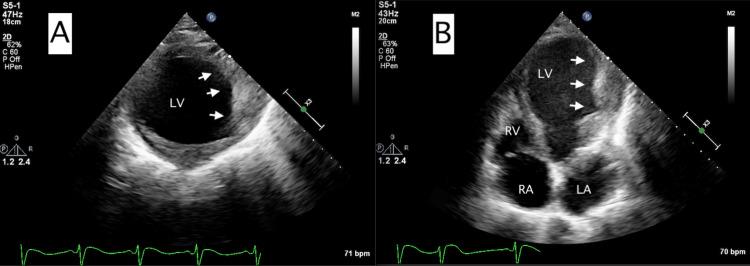
Transthoracic echocardiogram with thrombus formation in pseudoaneurysm There is severe left ventricular dilation with concentric remodeling of the left ventricle. Anterolateral pseudoaneurysm with an echogenic material suggestive of thrombus formation. A: Left ventricle short axis view with thrombus formation in the pseudoaneurysm (white arrows). B: Four-chamber view with thrombus formation in the pseudoaneurysm (white arrows). LV: left ventricle, LA: left atrium, RV: right ventricle, RA: right atrium.

A follow-up echocardiogram performed two weeks post LVPA diagnosis showed full thrombosis of the pseudoaneurysm (Figure 5), yet his LVEF remained critically low at < 15%. Given these findings and his clinical trajectory, the multidisciplinary heart team concluded that the patient was not a candidate for an implantable cardioverter defibrillator (ICD) due to the advanced stage of heart failure (New York Heart Association (NYHA) class 4D) and a limited life expectancy not meeting the criteria for expected survival beyond one year.

In light of the patient's deteriorating clinical status and limited therapeutic options, the palliative care team was engaged to guide end-of-life care planning. After thorough discussions regarding his prognosis and available treatments, the patient chose to transition to home hospice care, prioritizing comfort and quality of life. During his time in hospice, he continued to receive conservative medical management with the previously optimized medications. This supportive approach allowed him to spend his remaining time in a familiar environment, surrounded by his loved ones. He survived an additional five months under outpatient palliative care before succumbing peacefully at his home, demonstrating the profound impact of comprehensive, patient-centered care in advanced cardiac disease.

## Discussion

One of the most dramatic complications of AMI involves the tearing or rupture of acutely infarcted myocardium. Its diagnosis should be considered in post-myocardial infarction (MI) patients whenever there is severe hemodynamic instability or a sudden change in clinical condition. The clinical presentation of myocardial rupture depends upon the extent and site of rupture and is usually present in the first five days after MI [2-3]. Factors related to free-wall rupture include first MI (from lack of collateral blood supply), older adults (>70 years), women, hypertension during the acute phase of MI, use of corticosteroids or non-steroidal anti-inflammatory drugs, and missed or delayed reperfusion for an occlusive coronary MI. LVPA is a rare presentation of cardiac rupture, commonly described as an incomplete or contained rupture by scar tissue or adherent pericardium [1-3].

Based on a previous comprehensive analysis of 290 patients conducted through a systematic literature review, the most prevalent associations with LVPAs were MI (55%), surgical interventions (33%), and traumatic events (7%) [6]. LVPAs are caused mainly by inferior MIs about twice as frequently as anterior MIs, with a predominant posterior location (43%), followed by lateral, apical, inferior, anterior, and basal locations [1,6]. This is in line with irrigation areas linked to poor circulation. It has also been hypothesized that these locations relate to body-dependent pericardial adhesions developing in the recumbent, convalescing patients after infarction or pre-existing pericardial adhesions. Pre-existing pericardial heart disease may contribute to the latter, as may occur with HIV infection. The prevalence and incidence of pericardial heart disease in patients with HIV is elevated, most cases being asymptomatic and without an identifiable cause. A large autopsy and echocardiographic series involving 1139 patients with HIV infection revealed that the average incidence of pericardial disease was 21% [7]. Also, our current patient, with a prior history of cryptococcal meningitis, may implicate this fungal infection as a potential indolent cause of pericardial involvement, mostly when HIV-infected patients with cryptococcosis frequently present with disseminated disease.

In contrast to LVPAs, only 4% of true LVAs are found at the posterolateral or diaphragmatic surface. True aneurysms occur between 5-15% of patients with MI and are more commonly associated with large anteroapical infarctions [4]. In contrast to LVPA, a true ventricular aneurysm forms because of weakness rather than a rupture of the wall that typically bulges outward during systole, is thin, and contains all layers of the myocardium. In our current case, the myocardial rupture seems to occur in the margin between his post-MI anterolateral akinetic to the dyskinetic wall and his basal anterolateral contractile myocardium, suggesting an early LVA wall rupture.

It is noteworthy that LVPA poses a significantly higher risk of rupture progression and sudden death compared to true aneurysms. Since this patient was discharged on warfarin anticoagulation for management of paroxysmal atrial fibrillation (AF) and LVA thrombosis, combined with dual antiplatelet therapy (DAPT), including clopidogrel (indefinitely) and aspirin to complete 30 days according to clinical guidelines [8,9], this triple antithrombotic treatment (TAT) may have increased his bleeding risk and development of LVPA. Published data, clinical guidelines, and consensus statements [10] are available to establish increased bleeding risk with TAT in post-PCI populations with AF. However, this patient did not undergo PCI, and the true cardiovascular event reduction from dual antiplatelet treatment on top of warfarin is lower while exposing the patient to a higher risk of bleeding. Also, direct-acting oral anticoagulation (DOAC) for AF has a lower risk of bleeding than compared to warfarin. However, there is little data to support the use of DOAC for LV thrombosis. The use of clopidogrel over alternate P2Y12 inhibitors also offers a decreased risk of bleeding.

Urgent diagnostic and management strategies are paramount in treating these patients with LVPA. Despite the advancements in diagnostic techniques that facilitate early detection of LVPA, there remains a considerable risk of rupture ranging from 30% to 45% [1,6]. The precise underlying causes of LVPAs have yet to be fully elucidated. Typically, these abnormalities arise when a small, weakened section of the cardiac wall, often associated with a transmural infarction, ruptures gradually over an extended period. This rupture leads to a narrow opening, connecting the ventricular cavity to the potential space beneath the pericardium. Consequently, blood flows back and forth through this restricted orifice, eventually accumulating blood within the pericardial space or among pericardial adhesions. Because of this accumulation, an outward bulging occurs over the affected area. It is worth noting that similar weak protrusions in the heart wall can also be observed in true ventricular aneurysms, and since the clinical manifestations of both conditions lack specificity, distinguishing between them poses a diagnostic challenge. A study with a small participant pool investigated two-dimensional echocardiography to distinguish between patients with pseudoaneurysms and those with true aneurysms. The study employed a ratio of <0.5 between the size of the orifice and the size of the aneurysm sac to favor pseudoaneurysm [11], different from true aneurysms that commonly present with a broad base. However, relying solely on this measurement may prove insufficient in effectively distinguishing true LVAs from false ones [1,6], and is often very challenging. Table 1 summarizes multiple clinical and morphologic feature differences between LVA and LVPA.

**Table 1:** Clinical and feature differences between true and false ventricular aneurysms LVA: left ventricle aneurysm; LVPA: left ventricular pseudoaneurysm

Features:	LVA: True Aneurysm	LVPA: False Aneurysm
Incidence	5-15% of myocardial infarctions	Rare (<0.1%)
Pathophysiology	Full-thickness infarct, replaced by fibrotic tissue. Wall expansion and dyskinesia	Myocardial rupture into the pericardium
Location	Anteroapical (approximately 85%)	High
Risk of Rupture	Low (but higher in the early phase)	High
Treatment	Medical	Surgical (if symptomatic or early presentation)
Transthoracic echocardiogram diagnostic accuracy	Good	Limited
Neck	Broad, wide	Narrow
Wall	Myocardium	Thrombus and pericardium
Neck/sac ratio	0.9-1	<0.5
Complications	Mostly Asymptomatic, Heart failure, ventricular arythmias, thromboembolism; Rupture risk low (<5%)	Mostly symptomatic (non-specific), chest pain, dyspnea, heart failure, thromboembolism, arrhythmias, or sudden death. >10% asymptomatic. Rupture risk early post-myocardial infarction: high
Mortality	Six times higher than those without aneurysm	48% when managed medically, < 10% when managed with current improved surgical techniques

The diagnosis requires a high index of suspicion and often necessitates multiple complementary imaging tools. Transthoracic echocardiography (TTE) is a reasonable first test because it is noninvasive, affordable, and generally available for bedside use. Using the two-dimensional TTE, the vascularity of a suspected pseudoaneurysm can be examined by detecting to-and-fro Doppler flow, as the narrow neck and a wide aneurysmal sac (described above) are the hallmarks of a pseudoaneurysm on echocardiography [11]. Pulsed wave Doppler and color flow Doppler echocardiography have been used to detect high-velocity, turbulent, bidirectional flow between the left ventricle and pseudoaneurysm [12,13]. The usual blood flow enters the pseudoaneurysm sac during systole and returns to the LV in diastole. These techniques are instrumental when the location of the small connecting orifice with echocardiography is difficult. In the current case, the pseudoaneurysm neck is 2 cm wide and associated with severe LV systolic dysfunction, leading to stagnant blood flow and a slow swirling movement of blood between the LV and the pseudoaneurysm. For this reason, the absence of a turbulent flow through the neck of an aneurysm does not confirm a true LVA, making this finding specific LVPA but not that sensitive.

A conclusive diagnosis is made in only 26% of cases, so it is frequently nondiagnostic [1,12]. Transesophageal echocardiogram (TEE) allows enhanced detection of ventricular pseudoaneurysms up to 75%; however, this procedure is minimally invasive and more costly [14]. On cardiac magnetic resonance imaging (CMRI), the pseudoaneurysm's orifice shows a loss of epicardial fat [15]. CMRI is the best imaging modality to distinguish LVPAs from true aneurysms, and it has been reported to have a sensitivity of 100% and a specificity of 83% [16]. Its utilization is limited by issues surrounding its availability. However, in patients with prior infarction, it may be challenging to distinguish them from real aneurysms due to poor signal myocardium of infarcted tissues [15]. Cine MRI may offer further diagnostic capabilities by assessing blood flow turbulence in the heart chambers, one of the hemodynamic hallmarks of the pseudoaneurysm [15]. Cardiac CT, being more available than cardiac MRI, provides a great view of the LV myocardium, coronary arteries, and bypass grafts. However, it includes radiation and requires intravenous dye exposure [15]. Left ventriculography demonstrates the narrow neck connecting the ventricle to the pseudoaneurysm, in which contrast material remains for several beats after injection. This modality was previously considered the gold standard with a diagnostic accuracy of about 85% [17]. However, it is seldom used due to invasiveness and increased risk of thromboembolism or worsening rupture when performing ventriculography. A narrow orifice leading into a saccular aneurysm and the absence of collateral coronary arteries are two angiographic characteristics that might assist in detecting pseudoaneurysms [18]. When considering surgery, coronary angiography is typically required to determine whether concurrent bypass grafting is necessary [18]. Diagnosis may be missed when the radiograph is not perpendicular to the pseudoaneurysm, causing overlap with the left ventricle, or when insufficient contrast is employed [19].

LVPAs can manifest with dyspnea, congestive heart failure, chest pain, arrhythmias, thromboembolic events, and sudden cardiac death [6]. It is very similar to symptoms related to ischemic heart disease. In addition, >10% of people have asymptomatic presentations [6] or incidentally diagnosed upon imaging evaluations. The lack of clarity in the presentation may lessen the suspicion of this complication, leading to diagnostic delays. In a surgical registry of post-infarction LVPA that underwent surgical correction at Cleveland Clinic (from 1989-2002), the most common clinical presentation was heart failure symptoms in 73% and angina in 41%, with a median delay to diagnosis of 52 days (range, 2-268) after MI [20].

Although our patient had most of the above manifestations, he also presented with symptomatic sustained VT that was appropriately detected and treated by his WCD, protecting the patient from sudden cardiac death. In the 290 retrospective reviews with LVPA, approximately 3% of cases presented with sudden cardiac death [6]. The typical physical examination findings described is a fresh to-and-fro murmur [21]. However, the murmur may be challenging to distinguish from mitral regurgitation, and up to 30% of patients had no audible murmur. The presence and intensity of a murmur, like in a ruptured interventricular septum post-MI, will inversely correlate with the size of the defect and the residual LV systolic function. ECG findings are nonspecific and are present in up to 95% of patients, with 20% having ST-segment elevations. Cardiomegaly is the most prevalent chest X-ray abnormality. Occasionally, a chest X-ray may identify a new left ventricle border bulging configuration, which may prompt the investigation; however, these tests are unlikely to establish the diagnosis. Our patient's subsequent chest x-ray revealed an altered left ventricular configuration related to the anterolateral LVPA location (Figure 3). 

The leading cause of LVPA is MI [6]. Since early PCI became the standard of care for acute occlusive MI, the incidence of mechanical complications of AMI has decreased significantly, from 6% between 1977 and 1982 to 0.27% between 2003 and 2015 [4]. Our patient had a delayed presenting (>48h) anterolateral MI with subsequent development of a mid-apical LVPA. The patient did not undergo revascularization because performing a PCI on a completely occluded artery of >24 hours after a STEMI should be avoided in patients who are stable and do not show signs of severe ischemia or hemodynamic compromise. The Occluded Artery Trial (OAT) demonstrated that there was no notable distinction in the combined outcome of death, reinfarction, or severe heart failure (class IV) after an average follow-up period of 5.8 years when comparing patients who underwent PCI versus those who received medical treatment [22]. 

A comprehensive approach is required to manage LVPAs. LVPA has been categorized according to time of presentation post infarction, as acute (< 2 weeks after AMI), subacute, or chronic (>3 months after AMI) [15]. To further define the presence of myocardial viability, subsequent PET/CT was performed on his initial admission to assess if the patient benefited from invasive angiography for further revascularization. However, he was found with extensive scar tissue involving the infarcted area without viable myocardium.

Most researchers recommend surgical treatment of LVPA, especially when it is discovered in the first three months after AMI, when it is symptomatic, and when it is expanding or large (>3 cm) [23]. Active surgical management involving either patch closure or primary closure is widely regarded as the optimal and strongly recommended initial approach [6,23] since untreated pseudoaneurysms have a 30-45% risk of rupture [6], with the highest mortality (48%) occurring in the acute phase, within a median time of less than one week [6]. However, 16 patients who survived the acute phase remained alive at a median of 156 weeks, suggesting a relative stability of chronic pseudoaneurysms. Patients who underwent surgery had significant mortality rates (23%), as did those who received just medical care (46%) [6]. For most patients with pseudoaneurysms, improvements in surgical technique may have reduced the postoperative mortality rate to 10% [24]. For appropriate candidates, surgery is considered the treatment of choice. For those at a higher risk of experiencing complications and mortality after undergoing surgery, medical management is preferred. The risk of rupture had been thought to be as high as 46%, although advances in imaging have increased the detection of 'incidental' pseudoaneurysms in asymptomatic patients, possibly diluting this rupture risk. In two series published in 1998 and 2003, 10 and nine patients, respectively, with ventricular pseudoaneurysms who did not undergo surgery, did not rupture over an average follow-up of four years [25,26].

A preference for medical management has been suggested for cases involving chronic pseudoaneurysms measuring less than 3 mm in size [27]. The primary goal of this therapy is to reduce the enlargement of the pseudoaneurysm. Additionally, it aims to decrease ventricular wall stress by reducing afterload and minimizing the risk of thromboembolism, which are both crucial aspects of the treatment approach. For smaller pseudoaneurysms, percutaneous techniques can be employed as a viable treatment option with minimal risk of severe rupture, thromboembolism, or cardiac failure [28]. Among these approaches, septal occlusion devices have proven effective in closing the pseudoaneurysms through a percutaneous procedure [28]. The selection of the most suitable management strategy necessitates individualized care, considering the patient's overall clinical condition and the characteristics of the pseudoaneurysm. Collaboration between a multidisciplinary team is imperative to formulate an optimal management plan. Also, regular monitoring and long-term follow-up are essential for assessing the efficacy of the chosen interventions and addressing potential complications.

## Conclusions

LVPA is a rare but serious clinicopathologic entity that necessitates prompt diagnosis and treatment. Due to the nonspecific nature of the clinical presentation, a high index of suspicion is essential for accurate diagnosis. The risk of rupture is significant, as these pseudoaneurysms have a propensity for rapid growth. Early detection and treatment are crucial to prevent potentially life-threatening complications. TTE is an acceptable initial diagnostic tool, but further imaging modalities should be employed when uncertainty remains despite negative findings. Cardiac CT imaging, TEE, or MRI offer higher sensitivity for detecting LVPAs than TTE. However, LV angiography remains the gold standard for diagnosis and surgical planning. Surgical intervention is generally considered the preferred course of treatment for eligible individuals with LVPAs. Embracing an integrated and multidisciplinary approach is essential for optimizing patient outcomes and improving the long-term prognosis for individuals with LVPA.
